# Biotechnology entrepreneurship - where no research has gone before

**DOI:** 10.1186/1479-5876-8-102

**Published:** 2010-10-15

**Authors:** Michael L Salgaller, Francesco M Marincola

**Affiliations:** 1 thinkBiotech, LLC, Washington, DC 20009, USA; 2 Infectious Disease and Immunogenetics Section (IDIS) - Department of Transfusion Medicine, Clinical Center, National Institutes of Health, Bethesda, MD 20892. USA

## Editorial

Many researchers have a scientific interest in taking their translational studies from bench to bedside. They want to take their ideas and see how far they can go towards bringing medical solutions to as many patients as possible. However, it can be frustrating when the clinical development of their work is beyond the scope and capabilities of their university or hospital. Even for those institutions with research beds, studies are usually limited to small, pilot studies. Even for those institutions with the necessary financial support, the manufacturing, regulatory, and legal essentials are not in place to evolve such studies to larger, later-stage human trials. This is nothing new. What is new is the rising tide of researchers turning to the for-profit world. Either directly - by starting their own companies, or indirectly - by working with existing pharmaceutical and biotechnology companies, researchers are moving more and more technologies towards the marketplace. *Biotechnology Entrepreneurship: From Science to Solutions *provides a real-world introduction to starting and growing life science companies, as well as useful material for medical researchers interested in getting their technologies to as many patients as possible.

Increasingly, universities and medical research centers are making technology transfer and development - such as filing patents and establishing formal industry relationships - a factor in tenure and promotion evaluation; the new millennia version of publish or perish. The out-licensing and commercialization of life science technologies are becoming valued parts of the academic career ladder. For example, North Carolina State University tenure and promotion process includes technology transfer to industry and filing patents - as part of more general definition of generating, contributing to, or disseminating knowledge[1]. One study found a direct relationship between the granting of tenure and the type of industry partnership necessary for a therapy or device to evolve from bench to bedside[2].

On a related note, another growing trend is the number of scientists deciding to take a more active, hands-on role in technology development - either by serving a prominent role in companies exploiting their research, or deciding to become entrepreneurs and start their own companies. The decision to leave academia, or at least divert significant energy and time, to be an entrepreneur is too often made without sufficient information. Yet, this ignorance is largely the fault of the system - rather than the scientist. The number of universities offering MD/MBA or PhD/MBA combined programs is increasing. Still, since few graduate programs historically offered any business, legal, or financial courses in their curricula, the vast majority of active life scientists have any training in, or exposure to, entrepreneurship.

### Biotechnology Entrepreneurship

From Science to Solutions [3] provides a real-world introduction to starting and growing life science companies, as well as useful material for medical researchers interested in getting their technologies to as many patients as possible. Founding a company - or playing an active role (e.g., serving as scientific director or member of the scientific advisory board - translates to investing your time, energy, and (in some instances) money. Entrepreneurship is a decision fraught with potential peril[4]. Even if "tenure isn't what it used to be," it is many times more secure and less volatile than plunging into a start-up. For those technologies involving medical devices or diagnostics, the risk is slight lower since they have shorter times and price-tags to commercialization and revenues. However, therapeutics are more popular, and for these advancements the time-line to commercialization is long (usually > 5 years) and - while less costly than the often-quoted $1-1.7B for a single drug from big pharma [5] - still requires tens or hundreds of millions of dollars. Nonetheless, biotechnology or pharma entrepreneurship can be the most rewarding and challenging pursuits in a research career - and one of increasing interest and activity.

Thus, today's medical researchers are helping new therapies, devices, and diagnostics address unmet and underserved medical needs in record numbers and in record time. They are taking to understanding the business side of product development with the same zeal with which they first learned scientific principles. It is great to observe PhDs and MDs so readily admit and address (by leaving well-established comfort zone) their lack of understanding of that business side. Never afraid to ask challenging scientific questions, they are taking that "why not?" attitude to the for-profit world. Collaborations previously limited other academics are being expanded to encompass technology transfer, intellectual property, balance sheets, and even marketing. Titles such as "CEO" and "Founder" are becoming common additions to "professor" and "tenured." Scientists can be just as successful building companies as they are in building research programs. Oh, and by the way, entrepreneurship creates jobs and helps the economy as well ... not a bad by-product!
